# Numerical and Experimental Evaluation and Heat Transfer Characteristics of a Soft Magnetic Transformer Built from Laminated Steel Plates

**DOI:** 10.3390/s21237939

**Published:** 2021-11-28

**Authors:** Eduardo Cano-Pleite, Andrés Barrado, Néstor Garcia-Hernando, Emilio Olías, Antonio Soria-Verdugo

**Affiliations:** 1 Thermal and Fluids Engineering Department, Carlos III University of Madrid (Spain), Avda. de la Universidad 30, 28911 Leganés, Spain; ngarcia@ing.uc3m.es (N.G.-H.); asoria@ing.uc3m.es (A.S.-V.); 2 Electronic Technology Department, Carlos III University of Madrid (Spain), Avda. de la Universidad 30, 28911 Leganés, Spain; barrado@ing.uc3m.es (A.B.); olias@ing.uc3m.es (E.O.)

**Keywords:** heat transfer, modeling, simulation, transformer

## Abstract

The present work evaluates, both experimentally and numerically, the heat transfer characteristics of a 5 kW three-phase transformer built from laminated steel sheets. The transformer is operated at different powers, and its temperature distribution is monitored using 108 thermocouples. The experimental measurements are used firstly to determine the heat dissipated at the core and the windings of the transformer. This information is used as an input for a finite element numerical model, which evaluates the heat transfer characteristics of the transformer. The model proposed in this work simply solves the diffusion equation inside the transformer, accounting for the anisotropic thermal conductivity of the different components of the transformer, together with well-known correlations at its boundaries. The results reveal that the proposed numerical model can correctly reproduce the maximum temperature, the temperature distribution, and the time-evolution of the temperature at specific points of the transformer measured during the experimental campaign. These results are of great use for the subsequent development of transformers of the same type in lab-scale or industrial-scale size and reveal the applicability of simplified numerical models to accurately predict the heat transfer characteristics of this kind of transformers.

## 1. Introduction

Power transformers are essential components for power conversion and power delivery systems. Key points on the design and operation of such components are their efficiency, reliability, compactness, and cost, which are the main challenges in the development of new power electronic converters [1,2,3]. Soft magnetic materials for the development of transformers have been paid progressively more attention during the last few years. These materials allow improving the magnetic performance of the transformer and their efficient operation at specific voltages [1]. The literature regarding these materials, including detailed reviews of available soft magnetic materials, is not scarce [4,5,6,7,8]. Among them, Rodríguez-Sotelo et al. [4] presented an extensive state-of-the-art review on advanced ferromagnetic materials in power electronic converters. They presented both qualitative and quantitative data, which allows comparing the principal characteristics of these materials. Azuma et al. [5] presented a review of the recent progress in Fe-based amorphous and nanocrystalline soft magnetic materials. In their work, they achieved a smaller core loss for a lower-rated amorphous wound core transformer. Soft magnetic materials have also been investigated and reviewed for medium frequency [6] and high-frequency applications [7]. Novel laser techniques have also been proposed to treat the surface of soft magnetic materials, allowing to improve the performance of transformers and to reduce their overall losses [9]. Different laser parameters have been tested, allowing to reduce the core losses [10], modify the magnetic properties [11], and improve the transformer noise behavior [12]. Johnson et al. [10] observed that laser scribing can reduce core losses by as much as 20%. Laser treated Finemet was investigated by Zeleňáková et al. [11], revealing the influence of the density of laser scribed dotted lines on the domain structure and the shape of the hysteresis loops. In Lahn et al. [12], optimized laser conditions during laser domain material refinement are found to improve the transformer noise behavior.

Numerical methods are of great support for the evaluation of the thermal characteristics of transformers. They provide a useful yet simple tool to evaluate the temperature evolution in the transformer, which can suffer dramatic heating due to its losses that can severely impact its overall efficiency. The thermal evaluation of a transformer is usually done using analytical thermal-circuit models or more complex simulations involving Finite Element Method (FEM) and/or Computational Fluid Dynamics (CFD) [13,14,15,16,17,18,19,20,21,22,23,24]. Numerous articles are present in the literature devoted to analytical methods to predict the temperature distribution in transformers [14,15,16,17,18,19,20]. Regarding analytical models, Pierce [14] developed one of the first systems of finite difference equations to predict the hottest spot temperature in the windings of a ventilated transformer. The numerical model allows evaluating the effect of several dimensional parameters on the ratio between the hottest spot and the average winding temperature of the transformer. Elmoudi et al. [15] proposed a transformer thermal dynamic model based on fundamental heat transfer theory. Rahimpoor and Azizian [16] made use of finite differences to successfully mathematically model a cast-resin transformer. Yang et al. [17] used an adjustable degree-of-freedom numerical method to compute the temperature distribution of electrical devices, decreasing the computational time and system complexity. Allahbakshshi and Akbari [18] applied a new integration method as a novel approach to the usual methodology used in the literature for oil transformers [19,20].

As stated above, the numerical assessment of transformers is not only limited to analytical or finite difference methods. FEM and CFD are also used for the thermal evaluation of such systems. Eteiba et al. [21] used FEM to evaluate the temperature distribution in power transformers in a cast-resin dry type transformer. To that end, the heat generation was used as an input in a model in which isotropic material properties were used. The air surrounding the transformer was also included in the model. The results presented a reasonable accuracy when compared with the experimental outcome. A multiphysics FEM-CFD strategy was presented by Akbari and Rezaei-Zare [22] to simulate the hot-spot temperature in transformer bushings, obtaining a consistent temperature rise. CFD was also used by Yaman et al. [23] to thermally model a power transformer with a cabin and accurately model natural convection. The similarity between the experiments and the simulations verified the validity of the model. Different strategies have been also used to account for the anisotropic heat transfer characteristics of the windings and laminated core. Smolka and Nowak [24] used 2D numerical models to determine the cross-section conduction of the winding and analytical methods to determine the thermal conductivity of the windings in the direction along the wires and the anisotropic thermal conductivity of the laminated steel core. Pradhan and Ramu [25] presented a detailed estimation of the thermal inhomogeneity of the windings to evaluate the hottest spot temperature of a power transformer, whereas Li et al. [26] considered a simple multi-media thermal conductivity to determine the thermal conductivity of the windings in the radial and axial directions. In these works, the thermal conductivity in the wire direction is much larger than the thermal conductivity in the axial and radial directions. Similarly, the thermal conductivity of the core in the direction perpendicular to the sheets is smaller than the thermal conductivity in the plane of the sheet. Both analytical and FEM/CFD methods, in combination with analytical estimations of the material properties, have been demonstrated to provide an accurate description of the heat transfer characteristics of transformers. In fact, recently, Chen et al. [27] compared thermal-circuit modeling and finite element modeling for dry-type transformers, achieving similar results for both models.

The present work presents experimental measurements of a 5 kW three-phase transformer whose core is built of stack steel plates and makes use of soft-magnetic materials. Numerous thermocouples are used to monitor the temperature distribution in the transformer. A simple numerical model, solved using a finite element commercial software, is proposed to evaluate the heat transfer characteristics of the transformer. As a novelty, well-known heat transfer correlations are combined with simple analytical evaluations so that particularities of the transformer, such as the presence of the stack steel plates or the direction of the winding, are accounted for in terms of a non-isotropic thermal conductivity of the material, retaining at all times the simplicity of the model. The experimental measurements are also used to evaluate the losses of the transformer for different input powers, which are used as inputs for the numerical model. The results here presented serve to evaluate the validity of a robust yet simple numerical model to estimate the operation capabilities of complex transformers. 

## 2. Materials and Methods

### 2.1. Experimental Setup

**Figure 1:**
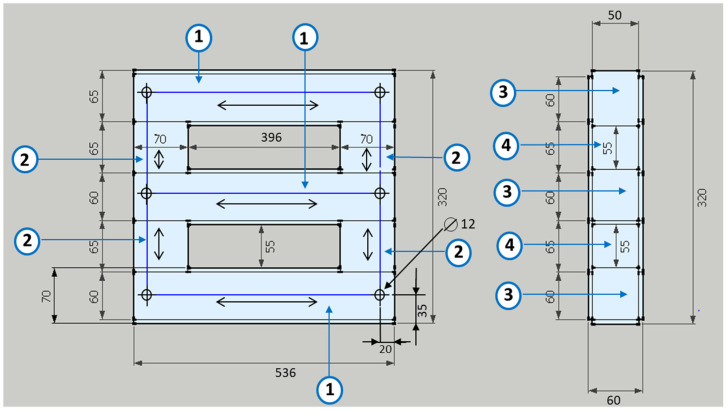
Main dimensions of the transformer. Dimensions of steel plates (mm): 1.60 × 536; 2.70 × 65; 3.70 × 536, 4.70 × 55.

A 5 kW three-phase transformer in D/Y configuration (380 V/220 V) has been developed for the numerical evaluation and heat transfer study. It consists of a natural air-cooled three-phase transformer designed to operate at 50 Hz. The core is made of laminated steel plates type 23MOH, and the total weight of the transformer, excluding hardware, is around 74.51 kg (57.71 kg core and 16.8 kg winding). The *B_max_* used to design the transformer was 1.5 T. The number of primary turns is 170, distributed in 2 layers, and the number of secondary turns is 288, distributed in 4 layers. The main transformer dimensions are shown in Figure 1.

**Figure 2:**
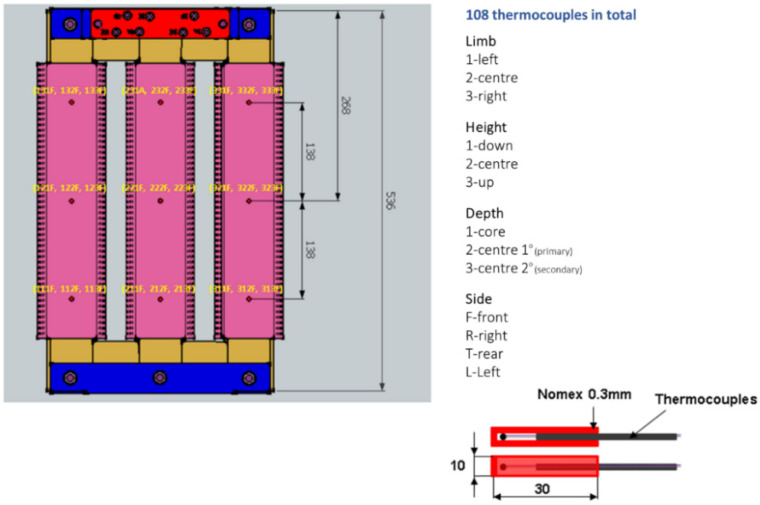
Temperature sensors, including the notation nomenclature for each of the sensors.

A total of 108 type K thermocouples were placed to measure the temperature at different transformer points during its operation. These thermocouples were placed at three different depths (stuck to the core, on the secondary winding face into the core, and on the primary winding face to outside), at three different heights (down, center, and up), and at each of the four faces of each limb. The nomenclature used to identify each of the 108 thermocouples includes four digits ABCD. The first digit, A, refers to the limb where the thermocouple is placed and can be 1 for the left, 2 for the center, or 3 for the right limb. The second digit, B, informs of the height at which the thermocouple was installed, being 1 for down (138 mm below the center of the limb), 2 for the center, or 3 for up thermocouples (138 mm above the center of the limb). The third digit, C, is related to the depth at which the thermocouple is located, and the possible values are 1 for sensors in direct contact to the core surface, 2 for sensors in the secondary winding, and 3 for sensors in the primary winding. Finally, the last digit, D, indicates the side where the measurement was carried out, using the values F for the front, R for the right, T for the rear, and L for the left sides. As an example, sensor 213F belongs to the center limb (**2**13F), down height (2**1**3F), primary winding (21**3**F), and the front side of the limb (213**F**). The detailed arrangement of these sensors and the nomenclature adopted for each measurement point is depicted in Figure 2.

**Figure 3:**
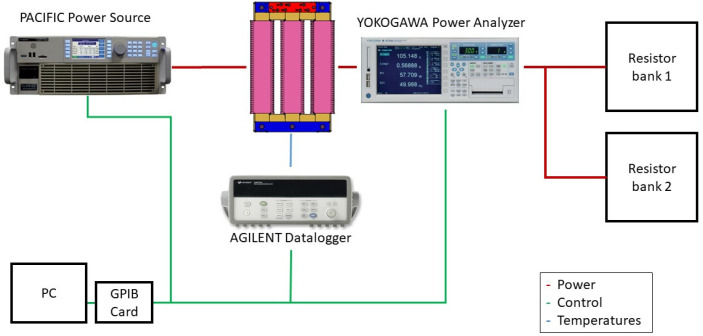
Final setup connection.

The final setup needed to conduct the measurements is composed of 8 elements: (i) 3-phase designed transformer, (ii) 3-phase power source: PACIFIC SmartSource 3150AFX, (iii) three-phase resistive load, (iv) datalogger: Agilent 34970A, (v) precision Power Analyzer: Yokogawa WT3000, (vi) thermal camera: NEC TH9100, (vii) national Instruments GPIB-USB-HS Card, and (viii) computer. Figure 3 shows the configuration of the different elements included in the setup.

Two 23 mm thick wooden planks and a 28 mm thick sheet of insulating material were placed under the transformer to prevent direct contact between the transformer and the ground. A thermocouple was placed below the insulating material to monitor the temperature of the floor, *T_floor_*, just below the transformer during the experiments.

**Figure 4:**
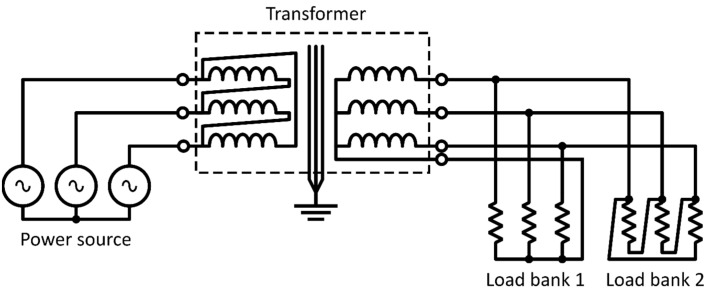
Electrical connection diagram of the transformer.

The primary configuration is in a delta and connected to the power source. The secondary winding is connected in a star configuration to load bank 1 and in a delta configuration to load bank 2. The electrical connection diagram is presented in Figure 4.

**Figure 5:**
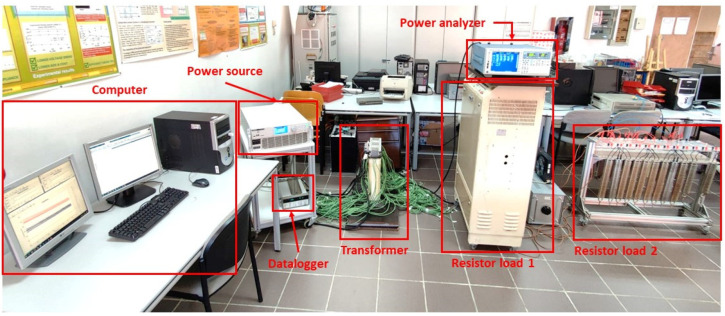
Global view of the total setup connection.

Figure 5 shows the different elements used in the experiments, which comprise the transformer itself, the power source, the power analyzer, the resistor loads, and the computer used to monitor the measurements.

### 2.2. Numerical Model

**Figure 6:**
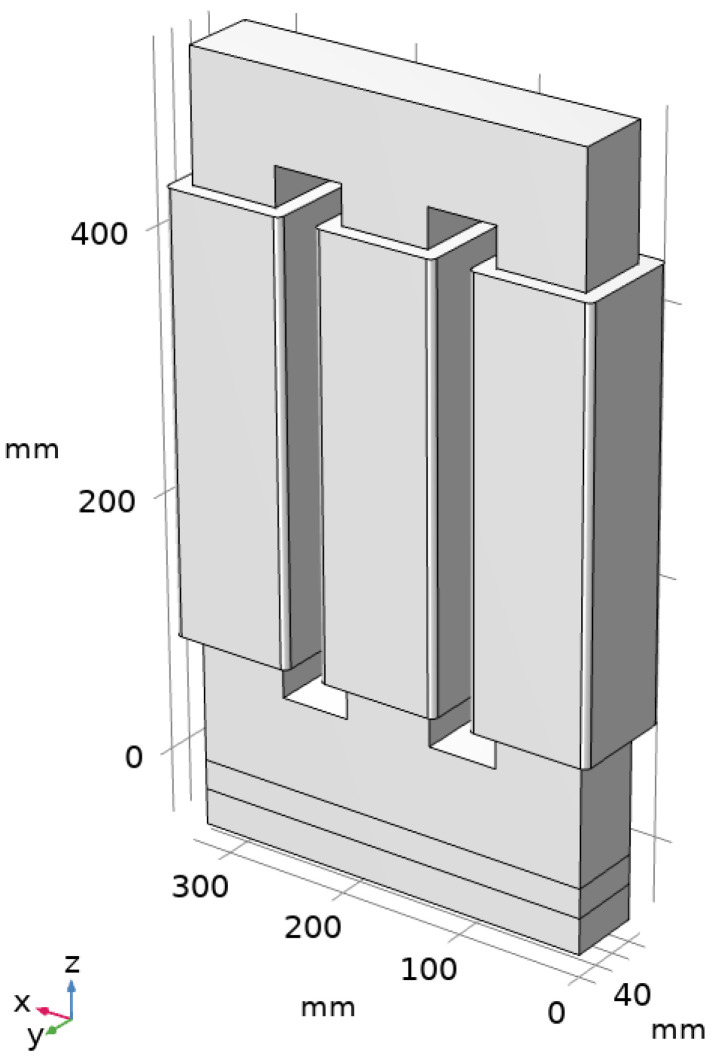
Three-dimensional numerical model.

The experimental setup described in Section 2.1 was replicated numerically using a three-dimensional finite element numerical model. The model, depicted in Figure 6, consists of a three-dimensional replica of the experimental system. In addition to the core and the windings of the transformer, the model also includes the wooden plate and the insulating material on which the prototype rests, which allows to establish the temperature at the base of the whole system, *T_floor_*.

COMSOL Multiphysics [28] was used for the numerical modeling of the transformer. The domain was discretized using a tetrahedral mesh with a maximum size of 5 mm and an average skewness of 0.7. A maximum time-step of 10 s was used for the transient simulations. Mesh size and time-step sensitivity analyses indicated that both the mesh and the time-step chosen were sufficiently fine to provide accurate results without largely increasing the computational cost of the simulations. An iterative Generalized Minimal Residual Method (GMRES) in combination with the Algebraic Multigrid (AMG) method is used to solve the model equations with a relative tolerance of 10^−3^.

#### 2.2.1. Constitutive Equations and Boundary Conditions

The numerical model proposed in this work only considers the solid bodies conforming the transformer. Heat is transferred in the three possible dimensions inside the solid bodies following the diffusion equation, which reads:

(1) $$\rho c_{p}\frac{\partial T}{\partial t}+\nabla \cdot q=Q_{gen}+Q_{dis}$$

where *T* is the temperature of the transformer at each spatial coordinate, *ρ* and *c_p_* represent the solid material density and specific heat, respectively, q=−k∇T is the heat rate conducted inside the solid body, *Q_gen_* denotes the heat generated inside the solid bodies per unit volume, which corresponds to the electromagnetic heating of the components of the transformer, and *Q_dis_* refers to the heat dissipated at the boundaries of the system per unit volume. For simplicity, no contact thermal resistances are considered between the different solid bodies that conform the transformer provided that a sensitivity analysis of the thermal contact resistance between the core and the windings resulted in maximum differences of the maximum temperature of the system below 3 °C. Furthermore, due to the symmetry of the system and the boundary conditions, two symmetry planes are employed. The first is a vertical symmetry plane (plane XZ in Figure 6) and the second symmetry plane considers the symmetry of the system regarding the central limb of the core (plane YZ in Figure 6). Therefore, the volume of the model is reduced to one-fourth, largely decreasing the computational cost of the simulations.

The transformer is surrounded by air at an experimentally measured room temperature, *T_a_*, and supported by a wooden plate, resting on an insulator plate in contact with another wooden plate over the laboratory floor. The whole transformer is initially at room temperature. The boundary conditions considered in the numerical model are natural convection and radiation at the external walls of the transformer and a prescribed boundary temperature at the base of the insulation plate, *T_floor_*, which was measured experimentally. The initial (IC) and boundary (BC) conditions can be expressed as follows:

(2) $$\mathrm{IC}:\;T\left(t=0\right)=T_{a}\left(t=0\right)$$

(3) $$\mathrm{BC}\;\mathrm{at}\;\mathrm{walls}:\;{q_{conv}}^{\prime \prime }+{q_{rad}}^{\prime \prime }=h_{conv}\cdot \left(T-T_{a}\left(t\right)\right)+\varepsilon \sigma \left(T^{4}-T_{a}{\left(t\right)}^{4}\right)$$

(4) $$\mathrm{BC}\;\mathrm{at}\;\mathrm{bottom}\;\mathrm{of}\;\mathrm{supporter}\;\mathrm{plate}:\;T=T_{floor}\left(t\right)$$

in which *h_conv_* refers to the natural convection coefficient and *ε* is an average surface emissivity used for the external surfaces of the transformer. The natural convection coefficient, *h_conv_*, was estimated using the widely known correlations of McAdams [29] and Churchill and Chu [30] as described below, whereas a nominal value of the average emissivity of the external surfaces of *ε =* 0.8 was selected based on the literature [16,21]. However, due to the incertitude associated with the actual emissivity of the boundaries of the transformer, different values of *ε* were used along this work to evaluate its effect on the steady-state temperature of the transformer. Furthermore, to simplify the numerical model and to avoid the use of surface-to-surface radiation conditions, the external boundary regions of the transformer that have a large view factor to other regions of the transformer, i.e., the regions of the core and windings in the internal part of each of the limbs, are not considered in ambient radiation. The duration of the experiments is above 10 h, which may cause significant variability in the laboratory conditions during the tests. Thus, in both Equations (3) and (4), the temperature of the ambient air *T_a_* and the bottom surface of the insulation plate *T_floor_* are time-dependent, and the values measured along the experiment are used as an input to the numerical model for validation of the transient heat transfer simulations. 

The heat dissipated by natural convection at the walls of the transformer can be divided into two contributions: those resorting from the horizontal surfaces, from which the correlation suggested by McAdams [29] is used for the hot surfaces of the components in upwards and downwards directions:

(5) $$h_{h,conv}=\{\begin{array}{c} \frac{k}{L}0.54\cdot {\mathrm{Ra}}_{L}^{1/4}\;\;\mathrm{if}\;\;10^{4}\leq {\mathrm{Ra}}_{L}\leq 10^{7}\\ \frac{k}{L}0.15\cdot {\mathrm{Ra}}_{L}^{1/3}\;\;\mathrm{if}\;\;10^{7}\leq {\mathrm{Ra}}_{L}\leq 10^{11} \end{array}\;\mathrm{if}\;\mathrm{the}\;\mathrm{plate}\;\mathrm{is}\;\mathrm{upwards}\;$$

(6) $$h_{h,conv}=\frac{k}{L}0.27\cdot {\mathrm{Ra}}_{L}^{1/4}\;\mathrm{if}\;10^{5}\leq {\mathrm{Ra}}_{L}\leq 10^{10}\;\mathrm{if}\;\mathrm{the}\;\mathrm{plate}\;\mathrm{is}\;\mathrm{downwards}$$

where *L* is the characteristic length of the horizontal surface, i.e., area divided by perimeter, and ${\mathrm{Ra}}_{L}$ is the Rayleigh number referred to this characteristic length and is defined as follows:

(7) $${\mathrm{Ra}}_{L}=\frac{g\beta \rho^{2}c_{p}\left(T-T_{a}\right)L^{3}}{\mu k}$$

where *g* is the gravity acceleration, and *β*, *ρ*, *c_p_*, *μ*, and *k* are the thermal expansion coefficient, the density, the specific heat, the dynamic viscosity, and the thermal conductivity of air, respectively. 

The contribution of the heat dissipated by external natural convection at the vertical boundaries of the model is evaluated using Churchill and Chu correlation for vertical plates [30], which reads:

(8) $$h_{v,conv}=\frac{k}{L}\left(0.825+\frac{0.387\cdot {\mathrm{Ra}}_{L}^{1/6}}{{\left(1+{\left(\frac{0.492}{\mathrm{Pr}}\right)}^{9/16}\right)}^{8/27}}\right){\;\mathrm{if}\;\mathrm{Ra}}_{L}>1$$

In this equation, *L* refers to the characteristic length of the appropriate vertical boundary, which, in this case, corresponds to the length of the plate in the vertical direction, and Pr is the Prandtl number for air. In the model presented here, it is considered that the vertical natural convection layers may interact which each other, even though their vertical surfaces are not in the same vertical plane, e.g., the vertical plates at the bottom region of the core and the vertical boundaries of the windings. Thus, it is assumed that these surfaces belong to the same vertical boundary in terms of natural convection and present a characteristic length that equals the whole transformer height.

#### 2.2.2. Materials and Physical Properties

**Figure 7:**
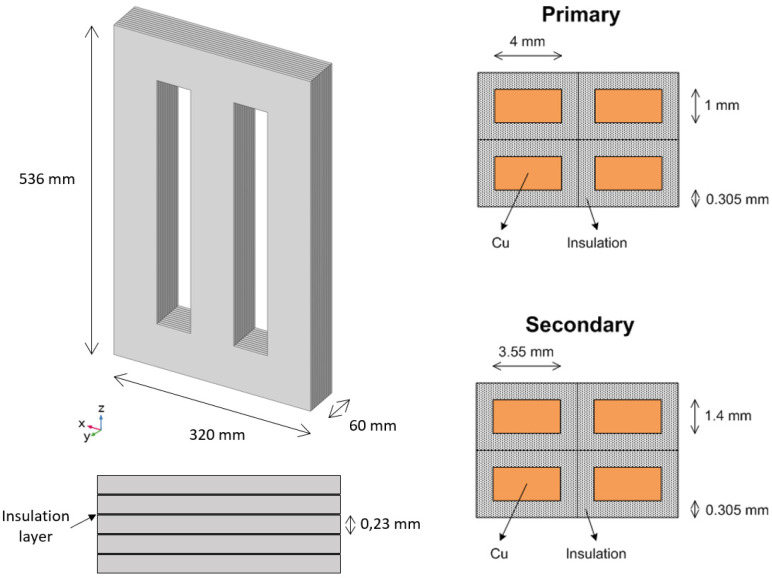
Schematic view of the core laminated sheet, including a detail of the sheet in the transversal direction and cross-section of the wiring in the primary and secondary. The scaling and the number of layers are schematic and do not correspond to reality.

The core is composed of a stack of several steel plates coated by an insulator. The thickness of each steel plate is 230 μm, and electric contact between the steel plates is prevented by a thin insulator layer. Figure 7 shows a schematic representation of the core laminated steel plates, in direction y in the figure, with the main core dimensions. Considering a value of the ratio of thicknesses between the insulator and the plates of 0.0175, the effective thermal conductivity of the core in each direction can be estimated according to [31,32], resulting in 51.1 W/(m·K) in the radial and angular directions (*x* and *z* in Figure 7) and 1.6 W/(m·K) in the axial direction (*y*-axis in Figure 7).

The winding consists of several wiring loops, including the copper conductor and the insulation, glass wool with a thickness of 305 μm, as schematized in Figure 7. Considering all the primary and secondary loops, the total thickness of the winding is 13.5 mm. The effective thermal conductivity of the winding can be estimated considering the conductivity in cylindrical coordinates. In the azimuthal direction, i.e., the wiring direction, the heat transfer is dominated by the high thermal conductivity of copper. Thus, the thermal conductivity in the wire direction (circumferential direction around the core limbs) can be assumed to be that of Cu, 380 W/(m·K). In contrast, the presence of the insulator may limit the thermal conductivity of the wiring in the radial and axial directions, i.e., perpendicular to the wires. The equivalent thermal conductivity in those directions can be estimated based on the distance between conductors and their characteristic length [31,32]. This results in thermal conductivity in the directions perpendicular to the wiring 20 times larger than the thermal conductivity of the insulating material. Therefore, considering the thermal conductivity of the insulating glass wool of 0.04 W/(m·K), the effective conductivity of the winding in the directions perpendicular to the wiring is 0.8 W/(m·K).

The total mass of the windings is 16.8 kg for the three limbs, considering only the mass of copper and neglecting the mass of glass wool due to its low density. However, the piling of the wires is not perfect, which requires the definition of an equivalent density of the windings that differs from that of the copper alone. The equivalent density of the windings can be estimated by means of their volume and the total mass, resulting in an equivalent density of 3992 kg/m^3^. Additionally, the density of the core was estimated by measuring the weight of the whole transformer, including the core and the fittings, and subtracting the winding mass, resulting in an equivalent density of the core of 9012 kg/m^3^.

Regarding the specific heat of the winding, since the mass of the glass wool used as insulation is negligible compared to the mass of copper, the specific heat of copper, 385 J/(kg·K), was used as a characteristic value for the winding. Thus, even though the limiting factor for heat transfer by conduction is the glass wool thickness, the limiting factor for heat absorbed is the copper conductor due to its higher mass. As a first approximation, the specific heat of the core was estimated to be that of the steel conforming it. However, comparison with the experimental measurements revealed that the heat capacity of the core, together with the insulation in between the different layers that form it, is around 738 J/(kg·K).

### 2.3. Heat Generation Due to Electromagnetic Losses

The heat generated by the device corresponds to the magnetic core losses and the windings Joule-effect losses, which are represented in the simulation through the volumetric generation term $Q_{gen}$ in Equation (1). Therefore, for the simulations, the heat generated is supplied in W/m^3^ and the volume of the core and the windings should be considered. 

The power analyzer used in the experimental facility provides the total losses generated in the experiments conducted with different input powers. However, the power distribution between winding losses and core losses was indirectly obtained. The procedure to separate the winding losses from the core losses is based on calculating the winding resistance considering the operating temperature. The first step was measuring the winding resistances at ambient temperature, obtaining 0.45 Ω for the primary and 0.15 Ω for the secondary. Then, the variation of the winding resistances with temperature was assumed to follow the following linear trend:

(9) $$R\left(T_{wind}\right)=R_{0}\cdot \left(1+\alpha_{Cu,0}\cdot \left(T_{wind}-T_{0}\right)\right)$$

where $T_{wind}$ is the approximate winding temperature, $T_{0}$ is the reference temperature, $R_{0}$ is the reference resistance at temperature $T_{0}$ and $\alpha_{Cu,0}$ is the copper temperature coefficient at the temperature $T_{0}$. In Equation (9), for $T_{0}=20\;℃$, $\alpha_{Cu,20\;℃}=0.00393\;K^{-1}$ and the reference winding resistances, measured at a temperature of 20 °C, are $R_{0,pr}=0.45\;\Omega \;$ and $R_{0,sec}=0.15\;\Omega \;$ for the primary and the secondary, respectively.

Since the exact temperature in each winding is not precisely known, the winding temperature was assumed to be between the temperatures measured on the core (core) surface and in the primary winding (pri). The expression used for this approximation is shown below:

(10) $$T_{wind}=T_{min,pri}+w\cdot \left(T_{max,core}-T_{min,pri}\right)$$

where $T_{max,core}$ is the maximum internal temperature, $T_{min,pri}$ is the minimum temperature registered at the primary winding, and w is a weighting coefficient between 0 and 1. A coefficient w = 0.4 was selected for the primary winding and w = 0.9 for the secondary winding, based on the location of the thermocouples.

With all the above, the following expressions are used to calculate the losses in the windings:

(11) $$P_{loss\_pr}=\left({\left(I_{rms,\;R}\right)}^{2}+{\left(I_{rms,\;S}\right)}^{2}+{\left(I_{rms,\;T}\right)}^{2}\right)\cdot \frac{R_{pr}}{3}$$

(12) $$P_{loss\_sec}=\left({\left(I_{rms,\;R}\right)}^{2}+{\left(I_{rms,\;S}\right)}^{2}+{\left(I_{rms,\;T}\right)}^{2}\right)\cdot R_{sec}$$

where *I_rms_* represents the root mean current of each of the R-S-T phases of the transformer, *R_pr_* and *R_sec_* are the resistances of the primary and secondary, respectively. Finally, *P_loss_pr_* and *P_loss_sec_* correspond, respectively, to the power losses in the primary and secondary, considering the primary is connected in delta and the secondary in star (see description of Figure 4). 

Once the winding losses at a given temperature were calculated, the core losses were determined by subtracting the winding losses from the total losses. Table 1 summarizes the different experimental cases analyzed, including the total losses in the whole transformer and the percentage of these losses corresponding to the core and the winding. The total power losses of the transformer increase proportional to the input power, due mainly to heat losses in the windings caused by the Joule effect. In contrast, the heat losses in the core are roughly uniform with the power input, increasing slightly for high power inputs.

## 3. Results and Discussion

The results obtained in this work are divided into two clearly distinguishable sections. The first section presents and analyses the results obtained from the experimental measurements. The results acquired from the numerical simulations are presented in the second section, together with a detailed validation of the numerical method under steady-state and transient conditions by comparison with the experimental results.

### 3.1. Experimental Results

From the 108 thermocouples included inside the three-phase transformer, a total of 30 thermocouples have been selected for the thermal analysis, plus two additional ones that measure the ambient temperature *T_a_* and the floor temperature *T_floor_*. This set of thermocouples show the thermal behavior of the transformer. 

The following criteria have been considered to choose the most representative measurement points, which are summarized in Table 2:

- The thermal behavior of the left and right limbs is similar. For that reason, only temperatures of the left and central limbs, i.e., T1xxx and T2xxx, were considered.
- The thermal behavior of the front and rear sides is also similar. Thus, only temperatures of the front side were evaluated (TxxxF).
- The temperature value reached at the intermediate depth (Txx2x), i.e., in the secondary winding, must be a value between the core surface and the primary winding temperatures. Hence, only temperatures on the core surface and the primary winding were selected (Txx1x and Txx3x).
- The temperature distribution in the center limb is symmetrical. Therefore, only the temperatures of the front and left of the center limb were chosen (T1xxF and T1xxL).

The electrical measurements were conducted once the transformer reached its steady-state operating temperature, which is also the maximum temperature registered. Over 15 h were needed to reach steady-state conditions in some cases. 

**Figure 8:**
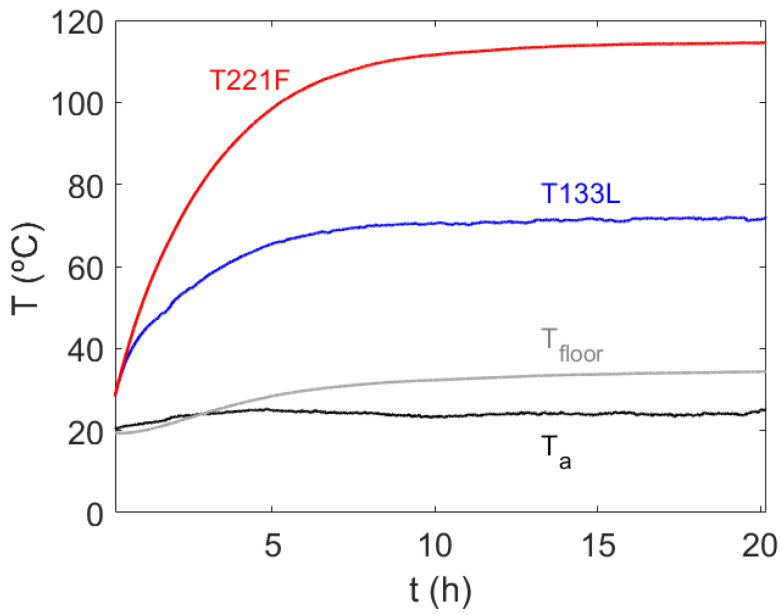
Time evolution of the maximum, minimum, wood bottom, and ambient temperature measured by the thermocouples in the system.

For each case of study, a set of data, waveforms, and thermal information were obtained and used to perform the theoretical calculations. The associated error of these measurements, based on the incertitude of the power analyzer is 0.02% of reading plus 0.04% of range. The signals of the thermocouples provided the temperature at different points in the transformer with an accuracy of ±1.1 °C. As a reference, Figure 8 shows the time evolution of the temperature measured by the thermocouples corresponding to the maximum (T221F) and minimum (T133L) temperature in the transformer for Case 6 (see Table 1), together with the value of the ambient temperature *T_a_* and the temperature at the base of the insulator situated below the transformer, *T_floor_*. Thus, all the rest of the temperatures measured during the experiment remain between the red and blue curves of Figure 8. All the registered temperature signals present an inverse exponential increase, reaching steady-state conditions after 15 h since the transformer was switched on. The heating time and the temperature were, as expected, different depending on the position of the temperature sensors. For instance, the minimum measured temperature corresponds to sensor T133L, which is situated on the left-hand side of the first limb, below the center (down location) of the primary winding, and reaches a steady temperature after around 10 h. The maximum measured temperature in the system occurs at the plane of symmetry of the transformer, on the core surface (T221F), reaching steady conditions around 15 h after the transformer is switched on. From a practical point of view, it is of great interest to demonstrate the capabilities of the model to predict this maximum temperature, as it will be the limiting operative factor of the transformer. Thus, this point will be further analyzed and compared with the outcome of the simulations in the subsequent sections. 

### 3.2. Numerical Results

**Figure 9:**
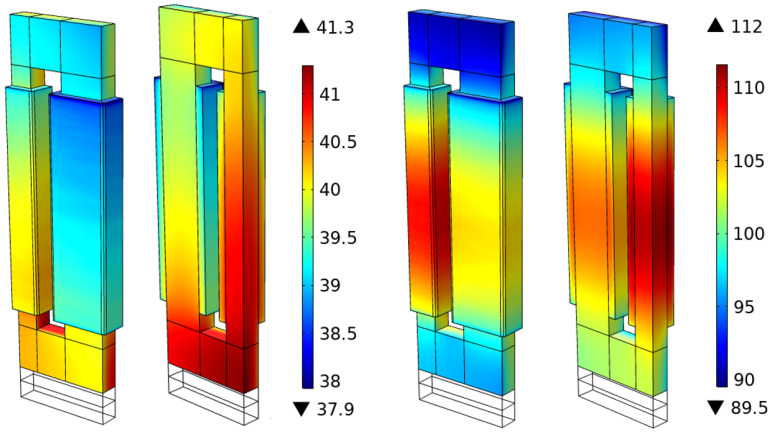
Snapshots of the simulated temperature distribution, in °C, of the transformer under steady-state conditions. Cases 1 (**left**) and 6 (**right**).

Figure 9 shows a snapshot of the temperature distribution in the transformer for Cases 1 and 6 under steady-state conditions, corresponding to a total heat dissipation of the transformer of 71.8 and 437.5 W, respectively. In the snapshots, the effect of both the higher electromagnetic losses in Case 6, together with the higher heat generation in the windings, can be observed. In both cases, the maximum temperature occurs in the symmetry plane near the center of the core. For Case 1, the maximum temperature is located closer to the bottom of the core due to the larger relative importance of non-symmetric dissipation in the vertical direction: natural convection occurs at the top plate of the transformer, whereas the bottom part of the transformer dissipates heat by conduction to the wood plate situated below it. However, for Case 6 in Figure 9, and the rest of the cases, the temperature distribution in the windings and the core clearly differs between the different cases, making the maximum temperature to occur in the vicinity of point 221F, that is, in the symmetry plane of the center limb, between the core and the windings. This difference between Case 1 and the rest of the cases is attributed to the different volumetric heat generation. Note that, in Case 1, 93.4% of the total heat generated occurs in the core, whereas only 23.1% of the total heat dissipation arises in the core for Cases 6.

**Figure 10:**
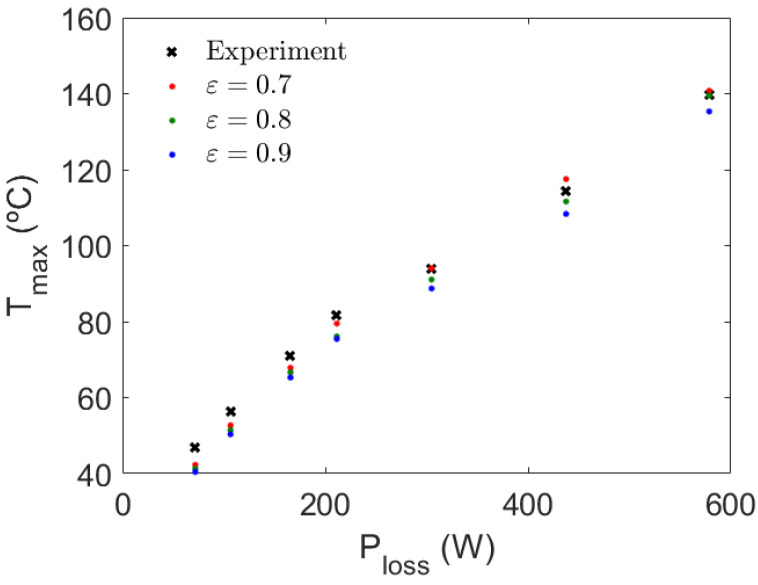
Comparison of the maximum temperature measured in the prototype and the numerical model estimation for the cases listed in Table 1 for various surface emissivity values.

In view of Figure 9, the maximum temperature in the system takes place on the symmetry plane YZ (see Figure 6), between the core and the winding. This location corresponds to thermocouple 221F, in accordance with the maximum temperature measured during the experimental procedure. Thus, from a practical point of view, it is of interest to monitor the temperature predicted by the simulation at this specific point where the maximum temperature of the prototype is located to compare it with the experimental outcome. The results of this comparison are presented in Figure 10 for all the cases listed in Table 1. In the figure, different values of the external surface emissivity of the materials (core and windings) are used due to the incertitude on their determination, confirming the appropriateness of the use of an average surface emissivity of 0.8 for the transformer boundaries, as stated in the specific literature [16,21]. In view of Figure 10, it can be concluded that the numerical model can predict with great accuracy the maximum temperature in the system for all the experimental cases tested, i.e., for a wide variability of the electromagnetic losses and heat dissipation distribution. The maximum difference between the experiments and the simulations, for an average surface emissivity of 0.8, is 6.5 °C and occurs for Case 1, while this difference progressively decreases for larger powers. The temperature differences between the model and the experimental results can be considered sufficiently low, given the simplicity of the model developed and the uncertainties attributed to the determination of the material properties and the simulation conditions.

Besides the estimation of the maximum temperature, the numerical model can also be used to predict the steady-state distribution of the temperature in the whole transformer, which is also of practical importance for the design and operation of these devices. Table 3 compares the experimental and numerically obtained temperature values at different measurement points in the transformer for Case 6, i.e., for a total heat dissipated due to electromagnetic losses of 437.5 W. The temperature values are depicted for thermocouples situated on the surface of the core, which is the most representative temperature for comparison. Again, the accuracy of the model is remarkable, with numerically predicted values of the temperature very close to those measured in the prototype. The more significant discrepancy between the experiments and the simulations is encountered for point 221L, with an absolute temperature difference of 6.6 °C, whereas for the rest of the points, an average deviation of only 2.2 °C was attained. In view of these results, the model has been tested to predict the temperature distribution inside the transformer under steady-state conditions.

**Figure 11:**
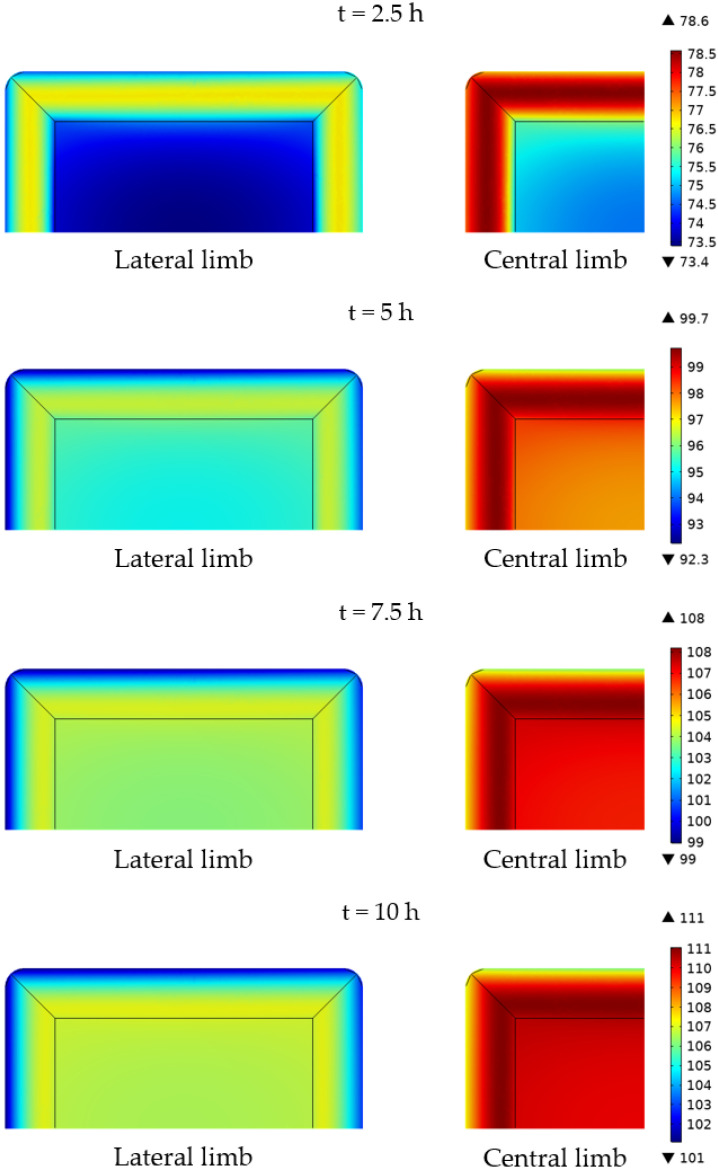
Temperature distribution, in °C, in a horizontal slice in the central section of the core and the windings for different time instants. Case 6.

In addition to the analysis of the performance of the simulations under the steady-state operation of the transformer, the accuracy of the thermal numerical model was also verified in terms of the time-dependent evaluation of the temperature of the system, that is, evaluating the transient heat transfer in the transformer. Figure 11 shows a horizontal slice of the temperature distribution of the core and the windings on the central section of the transformer evaluated at different time instants. The anisotropic conductivity of both the core and the windings can be especially noted in the figure during the first stages of the experiments. Note the uniformity of the temperature in the windings in the directions parallel to the core, which corresponds to the wiring loops direction. In this particular case, the heat generated in the core corresponds to the 24.3%, whereas the 75.7% of the total power losses occur in the windings. This unbalanced power loss promotes rapid heating of the windings. The high thermal conductivity of the windings in the azimuthal direction, together with the lower heat generation in the core that enhances conduction heat transfer from the windings to the core, and the convective and radiative heat transfer on the external surface of the windings, promote a larger temperature in the center of the windings during the first stages of the heating up process.

A similar though less observable anisotropic effect occurs in the core, in which the temperature is less uniformly distributed in the vertical direction in Figure 11, perpendicular to the laminated steel plates. The temperature distribution changes substantially during the first 7.5 h of the simulation. However, no significant differences in the temperature distribution are observed in the horizontal slice during the last 2.5 h, revealing the system has reached nearly steady-state conditions. 

In cases in which the percentage of power losses in the core is larger, the heating of the central region of the windings may be hindered by an increased heat transfer by conduction from the core to the windings, resulting in larger temperatures in the core that gradually decrease from the center of the system to the external surfaces, where heat is dissipated. In all cases, the lower thermal conductivity of the core in the transversal direction may cause the presence of hot spots in the central section of the core, especially if the heat generation mainly occurs in the core. Special attention should be paid in these cases, provided that possible experimental temperature measurements would be limited to the surface of the core or the core-winding interface, but not in the central sections of the core itself.

**Figure 12:**
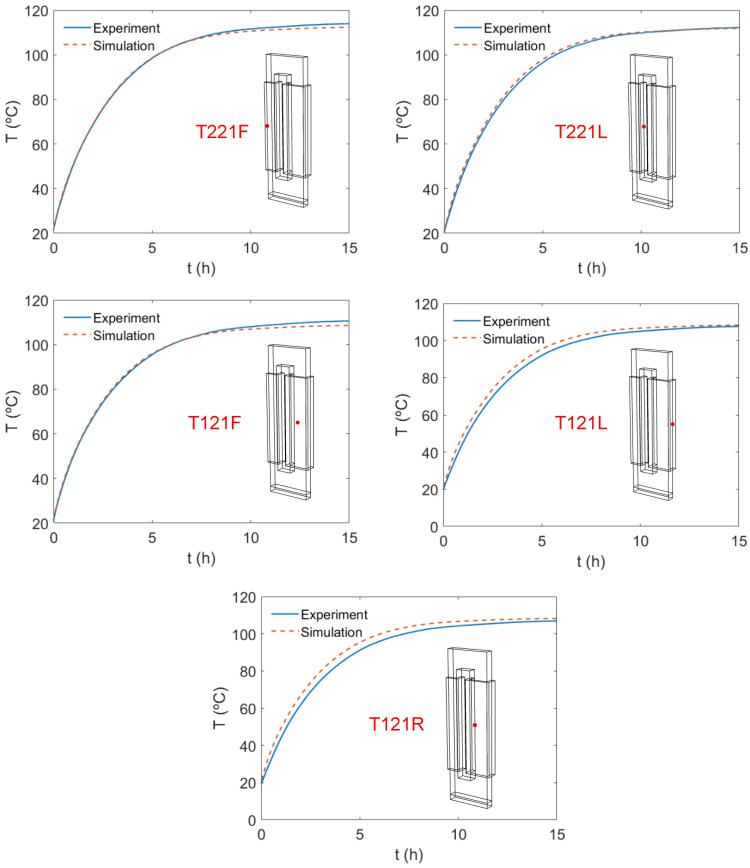
Time evolution of the temperature at different points of the transformer. Comparison between experiments and simulations. Case 6.

The time evolution of the temperature at different points of the transformer is evaluated in Figure 12, which shows the experimental and numerical evolution of the temperature obtained from the simulations and the experimentally measured values. In view of Figure 12, the model is capable of precisely determining the time evolution of the maximum temperature of the system, which accurately matches the heating sequence of the experiments. This also occurs for the rest of the points tested, located on the surface between the core and the winding. The transient results also allow to determine the transformer characteristic heating time, which can be arbitrarily defined as the average time required by the transformer to reach 90% of its maximum temperature at steady-state conditions, value at *t =* 15 h, as shown in Figure 12. Considering this definition, the simulation predicts an average characteristic heating time of the transformer of 5.4 h, which is very similar to the 5.9 h measured by the experiment. This again confirms the applicability of the model to predict not only the maximum temperature of the system but the time required to reach this maximum temperature.

Considering the detailed comparison of the transformer temperature distribution and evolution, both under steady-state and transient conditions, the predictions of the simple numerical model proposed, considering non-isotropic thermal conductivities for the core and windings, were found to be in excellent agreement with the experimental measurements conducted in the prototype. Therefore, the numerical model presented here was properly validated and can be used as a powerful tool to estimate the time evolution of temperature and its distribution in similar transformers with a very reduced computational cost.

## 4. Conclusions

The heat transfer characteristics of a novel 5 kW transformer made from laminated steel plates were studied in this work by means of experimental measurements combined with numerical simulations. The experimental facility was used to monitor the temperature evolution of the system, with a total of 108 thermocouples. The power measurements of the transformer at different operation conditions were employed to infer the power loss at the core and the windings of the system. This power loss was an input for the finite element numerical model, which is a three-dimensional replica of the transformer. As a novelty, the numerical model considers anisotropic material properties to account for the laminated steel plates from which the core is made and the non-isotropic conductivity of the winding. Well-established correlations are used to estimate the natural convection coefficients and the radiation dissipation to the surroundings of the transformer. The results reveal that the simple model proposed here is capable of accurately predicting both the steady-state temperature distribution of the transformer and the transient evolution of its temperature at different locations. Using an emissivity of external surfaces of 0.8, the highest deviation between the steady-state maximum temperature measured experimentally and that predicted by the numerical model is 6.6 °C, obtained for a case with a maximum temperature measured experimentally of 106.7 °C, i.e., for a temperature increase of 86.7 °C over ambient temperature, corresponding to a maximum deviation of 7.6%. This confirms the applicability of the model presented here for transformers with anisotropic properties and supports its use to predict the maximum temperature in the system, making it possible to anticipate the presence of hot-spots and malfunctioning operating regimes.

## Figures and Tables

**Table 1:** Cases simulated, including the total power input in the transformer and the power loss per unit volume in each of the components.

Case	Power Input, Transformer (W)	Total Power Losses (W)	Percentage of Power Losses in the Core (%)	Power Losses per Unit Volume—Core (kW/m^3^)	Power Losses per Unit Volume—Winding (kW/m^3^)
1	1205.5	71.8	94.3	8.8	0.96
2	4202.1	106.6	62.0	8.6	9.5
3	6547.3	165.7	39.4	8.5	23.6
4	7647.5	211.4	32.4	8.9	33.6
5	9399.7	304.8	27.0	10.7	52.3
6	11,160.3	437.5	24.3	13.9	77.9
7	12,534.2	578.7	23.1	17.4	104.7

**Table 2:** Location of the thermocouples.

		Left Limb			Centre Limb	
		Front	Left	Right	Front	Left
**Down**	**Core**	T111F	T111L	T111R	T211F	T211L
	**Centre2**	T113F	T113L	T113R	T213F	T213L
**Centre**	**Core**	T121F	T121L	T121R	T221F	T221L
	**Centre2**	T123F	T123L	T123R	T223F	T223L
**Up**	**Core**	T131F	T131L	T131R	T231F	T231L
	**Centre2**	T133F	T133L	T133R	T233F	T233L

**Table 3:** Temperature values under steady-state conditions for different points in the transformer. Comparison between experimental and numerical results.

Position	*T_sim_* (°C)	*T_exp_* (°C)	Deviation (°C)
231F	103.3	104.0	0.7
221F	111.2	114.2	3.0
211F	106.7	104.0	2.7
231L	103.3	104.2	0.9
221L	111.2	112.5	1.3
211L	106.7	100.1	6.6
131F	100.6	100.4	0.2
121F	107.0	110.9	3.9
111F	103.8	100.6	3.2
131L	100.7	99.2	1.5
121L	106.8	107.9	1.1
111L	103.9	101.4	2.5
131R	100.7	99.8	0.9
121R	106.8	107.2	0.4
111R	103.9	98.2	5.7

## Data Availability

Not applicable.
